# Supplementary material for: Straw-Enhanced Soil Bacterial Robustness via Resource-Driven Niche Dynamics in Tea Plantations, South Henan, China
Source: Microorganisms. 2025 Apr 6;13(4):832. doi: 10.3390/microorganisms13040832 (PMC12029857; doi:10.3390/microorganisms13040832)
Supplement: Supplementary file 1 [file microorganisms-13-00832-s001.zip › Table S2.pdf]

Table S2. The characteristics of the straw applied in the tea plantations

Straw types	N g/kg	P_2O_5 g/kg	Cellulose (%)	Hemicellulose (%)	Lignin (%)
Wheat	5.0~6.7	2.0~3.4	37.0~41.0	26.0~31.0	13.0~15.0
Rice	6.3~8.5	1.1~2.9	31.0~45.0	23.0~28.0	12.0~14.0
