# Supplementary material for: Straw-Enhanced Soil Bacterial Robustness via Resource-Driven Niche Dynamics in Tea Plantations, South Henan, China
Source: Microorganisms. 2025 Apr 6;13(4):832. doi: 10.3390/microorganisms13040832 (PMC12029857; doi:10.3390/microorganisms13040832)
Supplement: Supplementary file 1 [file microorganisms-13-00832-s001.zip › Table S3.pdf]

Table S3. Soil properties of different samples

	pH	EC ($ms \cdot cm^{-1}$)	SOM ($g \cdot kg^{-1}$)	AP content ($mg \cdot kg^{-1}$)	NH_4^--N content ($mg\ kg^{-1}$)	NO_3^--N content ($mg\ kg^{-1}$)	WC %	Al content ($mg\ kg^{-1}$)	Ca content ($mg\ kg^{-1}$)
CK1	6.25	143.3	22.0	9.56	15.1	1.35	19.6	10037	2192
CK2	6.36	77.8	22.0	9.80	20.7	0.91	23.5	18269	2751
CK3	6.04	94.2	25.5	9.08	18.8	1.24	19.9	15310	4143
CK4	5.69	121.8	30.6	8.37	24.0	1.35	18.2	16237	3498
CK5	5.86	103.1	24.9	9.56	21.0	1.41	24.7	5826	667
CK6	6.40	154.2	20.1	8.37	16.9	1.88	23.5	1023	13
CK7	5.56	114.2	31.9	8.37	15.7	1.46	28.9	9457	925
CK8	5.81	102.2	27.5	7.65	14.5	1.61	30.5	11675	1121
CK9	5.60	121.4	27.9	7.65	19.5	0.84	33.3	9430	1904
CK10	5.98	158.1	21.7	14.34	8.3	1.31	22.5	15274	4252
CK11	5.61	138.1	18.1	12.19	16.6	0.87	23.2	8427	1036
CK12	5.32	217.0	20.3	12.43	26.9	1.31	20.8	12548	2488
S1	6.03	161.7	24.2	8.37	16.2	0.88	23.5	14939	4373
S2	6.28	78.2	25.2	8.37	13.2	0.47	26.3	14347	3480
S3	6.14	133.2	25.1	7.89	14.7	1.55	22.9	17303	4727
S4	6.17	76.3	22.6	7.17	14.2	0.44	20.5	18790	5248
S5	6.36	55.1	24.4	8.13	15.9	1.61	25.0	6254	889
S6	6.13	148.0	25.9	8.60	15.0	1.21	27.9	10615	687
S7	6.11	133.0	22.8	8.13	17.0	0.84	23.8	10453	974
S8	6.00	188.9	25.3	8.37	15.3	1.44	26.6	9378	609
S9	6.05	91.6	29.7	7.41	7.4	1.44	40.4	11434	680
S10	6.30	97.4	24.6	7.17	16.6	1.42	34.4	12459	878

S11	6.45	101.7	26.3	7.17	17.6	1.14	37.7	9439	1408
S12	6.18	103.2	23.7	6.45	28.6	0.85	34.8	25534	3002

Electrical conductivity (EC), Soil organic matter (SOM), Available phosphorus content (AP), Soil gravimetric water content (WC).
