# Supplementary material for: Straw-Enhanced Soil Bacterial Robustness via Resource-Driven Niche Dynamics in Tea Plantations, South Henan, China
Source: Microorganisms. 2025 Apr 6;13(4):832. doi: 10.3390/microorganisms13040832 (PMC12029857; doi:10.3390/microorganisms13040832)
Supplement: Supplementary file 1 [file microorganisms-13-00832-s001.zip › Table S4.pdf]

Table S4 The community composition of dominant bacteria

Taxa level	Taxonomy	CK (%)	S (%)
Phylum	Acidobacteriota	19.7~36.6	20.9~29.0
	Actinomycetota	3.7~21.0	2.3~8.8
	Bacteroidota	1.0~7.8	1.9~13.4
	Chloroflexota	6.1~24.7	6.6~20.8
	Gemmatimonadota	1.7~9.0	2.2~8.5
	Myxococcota	1.1~8.6	2.3~9.3
	Nitrospirota	2.4~2.5	1.2~2.9
	Planctomycetota	3.4~7.3	4.6~8.0
	Pseudomonadota	18.2~27.2	16.6~26.4
Top 10 family	Incertae_Sedis_o_Subgroup_2	0.4~11.9	0.5~8.0
	Incertae_Sedis_o_Terriglobales	1.7~13.7	0.9~4.6
	Pyrinomonadaceae	0.1~8.4	0.6~4.6
	Incertae_Sedis_o_Vicinamibacterales	1.1~6.3	3.1~5.9
	Vicinamibacteraceae	0.2~4.5	1.5~4.6
	Incertae_Sedis_o_Gaiellales	0.8~15.3	0.5~2.5
	Anaerolineaceae	0.8~6.2	1.8~8.8
	Gemmatimonadaceae	1.7~8.3	2.1~8.4
	Xanthobacteraceae	1.9~4.8	2.0~4.2
	Nitrosomonadaceae	1.8~7.3	2.3~7.0
