# Supplementary material for: Straw-Enhanced Soil Bacterial Robustness via Resource-Driven Niche Dynamics in Tea Plantations, South Henan, China
Source: Microorganisms. 2025 Apr 6;13(4):832. doi: 10.3390/microorganisms13040832 (PMC12029857; doi:10.3390/microorganisms13040832)
Supplement: Supplementary file 1 [file microorganisms-13-00832-s001.zip › Table S5.pdf]

Table S5. The biomarker information between CK and S based on the linear discriminant analysis (LDA) threshold (LDA score > 3.0, *P* < 0.05)

Treatment	Taxa	*LDA Score*	***P***
CK	p__Acidobacteriota, c__Acidobacteriae	-4.47	0.028
	p__Acidobacteriota, c__Acidobacteriae, o__Terriglobales	-4.29	0.028
	p__Chloroflexota, c__Ktedonobacteria, o__Ktedonobacterales	-4.12	0.043
	p__Chloroflexota, c__Ktedonobacteria, o__Ktedonobacterales, f__JG30-KF-AS9	-4.05	0.040
	p__Chloroflexota, c__Ktedonobacteria, o__Ktedonobacterales, f__JG30-KF-AS9, g__Incertae_Sedis, s__uncultured_bacterium	-4.03	0.026
	p__Pseudomonadota, c__Gammaproteobacteria, o__Burkholderiales, f__Nitrosomonadaceae, g__Ellin6067	-3.63	0.017
	p__Acidobacteriota, c__Acidobacteriae, o__Terriglobales, f__Incertae_Sedis, g__Incertae_Sedis, s__uncultured_forest	-3.31	0.023
	p__Bacteroidota, c__Bacteroidia, o__Chitinophagales, f__Chitinophagaceae	-3.28	0.038
	p__Chloroflexota, c__Ktedonobacteria, o__Ktedonobacterales, f__Ktedonobacteraceae	-3.28	0.026
	p__Candidatus_Eremiobacterota	-3.19	0.041
	p__Candidatus_Eremiobacterota, c__Eremiobacteria	-3.19	0.041
	p__Actinomycetota, c__Actinobacteria, o__Frankiales, f__Acidothermaceae	-3.15	0.040
	p__Actinomycetota, c__Actinobacteria, o__Frankiales, f__Acidothermaceae, g__Acidothermus	-3.15	0.040
	p__Pseudomonadota, c__Gammaproteobacteria, o__Burkholderiales, f__SC-I-84	-3.09	0.035
	p__Actinomycetota, c__Actinobacteria, o__Frankiales, f__Acidothermaceae, g__Acidothermus, s__uncultured_bacterium	-3.07	0.028
	p__Acidobacteriota, c__Acidobacteriae, o__Terriglobales, f__Incertae_Sedis, g__Incertae_Sedis, s__uncultured_Acidobacteriaceae	-3.03	0.040

Treatment	Taxa	*LDA Score*	***P***
S	p__Chloroflexota, c__Anaerolineae	4.11	0.038
	p__Chloroflexota, c__Anaerolineae, o__Anaerolineales	3.99	0.018
	p__Chloroflexota, c__Anaerolineae, o__Anaerolineales, f__Anaerolineaceae	3.99	0.018
	p__Pseudomonadota, c__Gammaproteobacteria, o__Burkholderiales, f__Nitrosomonadaceae, g__MND1	3.83	0.021
	p__Bacteroidota, c__Bacteroidia, o__Cytophagales	3.8	0.028
	p__Bacteroidota, c__Bacteroidia, o__Cytophagales, f__Microscillaceae	3.79	0.028
	p__Acidobacteriota, c__Vicinamibacteria, o__Vicinamibacterales, f__Vicinamibacteraceae	3.71	0.033
	p__Bacteroidota, c__Bacteroidia, o__Cytophagales, f__Microscillaceae, g__Chryseotalea	3.6	0.030
	p__Planctomycetota, c__Planctomycetes, o__Pirellulales, f__Pirellulaceae, g__Pirellula	3.53	0.005
	p__Nitrospirota	3.49	0.028
	p__Planctomycetota, c__Planctomycetes, o__Pirellulales	3.41	0.033
	p__Planctomycetota, c__Planctomycetes, o__Pirellulales, f__Pirellulaceae	3.41	0.033
	p__Nitrospirota, c__Nitrospiria	3.41	0.043
	p__Nitrospirota, c__Nitrospiria, o__Nitrospirales	3.41	0.043
	p__Nitrospirota, c__Nitrospiria, o__Nitrospirales, f__Nitrospiraceae	3.41	0.043

Treatment	Taxa	*LDA Score*	***P***
	p__Nitrospirota, c__Nitrospiria, o__Nitrospirales, f__Nitrospiraceae, g__Nitrospira	3.41	0.043
	p__Chloroflexota, c__Anaerolineae, o__Anaerolineales, f__Anaerolineaceae, g__Incertae_Sedis, s__uncultured_soil	3.38	0.033
	p__Planctomycetota, c__OM190, o__Incertae_Sedis, f__Incertae_Sedis, g__Incertae_Sedis, s__uncultured_bacterium	3.29	0.022
	p__Myxococcota, c__Polyangiia, o__Polyangiales, f__BIrii41	3.26	0.024
	p__Acidobacteriota, c__Vicinamibacteria, o__Subgroup_17	3.26	0.002
	p__Nitrospirota, c__Nitrospiria, o__Nitrospirales, f__Nitrospiraceae, g__Nitrospira, s__uncultured_Green	3.25	0.009
	p__Planctomycetota, c__Planctomycetes, o__Pirellulales, f__Pirellulaceae, g__Pirellula, s__uncultured_bacterium	3.24	0.004
	p__Pseudomonadota, c__Gammaproteobacteria, o__Burkholderiales, f__Rhodocyclaceae	3.21	0.043
	p__Acidobacteriota, c__Subgroup_5	3.18	0.017
	p__Bacteroidota, c__Bacteroidia, o__Cytophagales, f__Microscillaceae, g__Chryseotalea, s__uncultured_bacterium	3.12	0.014
	p__NB1-j	3.12	0.019
	p__Chloroflexota, c__Chloroflexia, o__Chloroflexales	3.12	0.021
	p__Planctomycetota, c__Pla4_lineage	3.11	0.004
	p__Chloroflexota, c__Chloroflexia, o__Chloroflexales, f__Roseiflexaceae	3.1	0.021
	p__Myxococcota, c__Polyangiia, o__Haliangiales, f__Haliangiaceae, g__Incertae_Sedis, s__uncultured_bacterium	3.09	0.030

**Treatment**	**Taxa**	***LDA Score***	***P***
	p__Chloroflexota, c__Chloroflexia	3.08	0.043
	p__Acidobacteriota, c__Subgroup_5, o__Incertae_Sedis, f__Incertae_Sedis, g__Incertae_Sedis, s__uncultured_bacterium	3.06	0.011
	p__Pseudomonadota, c__Alphaproteobacteria, o__Dongiales	3.06	0.024
	p__Pseudomonadota, c__Alphaproteobacteria, o__Dongiales, f__Dongiaceae	3.06	0.024
	p__Pseudomonadota, c__Alphaproteobacteria, o__Dongiales, f__Dongiaceae, g__Dongia	3.06	0.024
	p__Acidobacteriota, c__Subgroup_25	3.05	0.022
	p__Myxococcota, c__Myxococcia	3.03	0.019
	p__Myxococcota, c__Myxococcia, o__Myxococcales	3.03	0.019
	p__Planctomycetota, c__Pla4_lineage, o__Incertae_Sedis, f__Incertae_Sedis, g__Incertae_Sedis, s__metagenome	3.01	0.005
	p__RCP2-54, c__Incertae_Sedis, o__Incertae_Sedis, f__Incertae_Sedis, g__Incertae_Sedis, s__uncultured_Desulfuromonadales	3.01	0.005
