# Supplementary material for: Straw-Enhanced Soil Bacterial Robustness via Resource-Driven Niche Dynamics in Tea Plantations, South Henan, China
Source: Microorganisms. 2025 Apr 6;13(4):832. doi: 10.3390/microorganisms13040832 (PMC12029857; doi:10.3390/microorganisms13040832)
Supplement: Supplementary file 1 [file microorganisms-13-00832-s001.zip › Table S6.pdf]

Table S6. The within/between-Module connectivity information of the keystone taxa of CK and S

Treatment	Sequence	Taxa	Z_i	P_i	Roles
CK	ASV4088	p__Acidobacteriota, c__Acidobacteriae, o__Bryobacterales, f__Bryobacteraceae, *g__Bryobacter*	5.25	0.00	Module hubs
	ASV8707	p__Acidobacteriota, c__Acidobacteriae, o__Subgroup_2, f__Incertae_Sedis, *g__Incertae_Sedis*	4.58	0.00	Module hubs
	ASV56288	p__Acidobacteriota, c__Acidobacteriae, o__PAUC26f, f__Incertae_Sedis, *g__Incertae_Sedis*	3.19	0.00	Module hubs
	ASV35430	p__Acidobacteriota, c__Vicinamibacteria, o__Vicinamibacterales, f__Vicinamibacteraceae, *g__Incertae_Sedis*	2.91	0.00	Module hubs
	ASV35834	p__Acidobacteriota, c__Acidobacteriae, o__Bryobacterales, f__Bryobacteraceae, *g__Bryobacter*	3.64	0.00	Module hubs
	ASV3383	p__Acidobacteriota, c__Vicinamibacteria, o__Vicinamibacterales, f__Incertae_Sedis, *g__Incertae_Sedis*	3.64	0.00	Module hubs
	ASV12759	p__Actinomycetota, c__Thermoleophilia, o__Gaiellales, f__Incertae_Sedis, *g__Incertae_Sedis*	2.99	0.00	Module hubs
	ASV23044	p__Actinomycetota, c__Acidimicrobiia, o__Microtrichales, f__Ilumatobacteraceae, *g__CL500-29_marine_group*	3.83	0.00	Module hubs
	ASV11962	p__Actinomycetota, c__Thermoleophilia, o__Gaiellales, f__Incertae_Sedis, *g__Incertae_Sedis*	-0.65	0.63	Connectors
	ASV51081	p__Myxococcota, c__Polyangiia, o__Haliangiales, f__Haliangiaceae, *g__Incertae_Sedis*	2.97	0.00	Module hubs
	ASV18106	p__Chloroflexota, c__Anaerolineae, o__Anaerolineales, f__Anaerolineaceae, *g__Incertae_Sedis*	3.18	0.07	Module hubs
	ASV42541	p__Chloroflexota, c__Anaerolineae, o__Anaerolineales, f__Anaerolineaceae, *g__UTCFX1*	2.91	0.00	Module hubs

Treatment	Sequence	Taxa	Z_i	P_i	Roles
	ASV33265	p__Chloroflexota, c__Anaerolineae, o__Anaerolineales, f__Anaerolineaceae, *g__UTCFX1*	2.97	0.06	Module hubs
	ASV14402	p__Chloroflexota, c__Anaerolineae, o__Aggregatilineales, f__Aggregatilineaceae, *g__Incertae_Sedis*	2.91	0.48	Module hubs
	ASV17372	p__Gemmatimonadota, c__Gemmatimonadia, o__Gemmatimonadales, f__Gemmatimonadaceae, *g__Incertae_Sedis*	2.97	0.00	Module hubs
	ASV17330	p__Myxococcota, c__Polyangiia, o__Haliangiales, f__Haliangiaceae, *g__Incertae_Sedis*	3.18	0.00	Module hubs
	ASV47351	p__Planctomycetota, c__Phycisphaerae, o__Tepidisphaerales, f__CPla-3_termite_group, *g__Incertae_Sedis*	5.02	0.05	Module hubs
	ASV41380	p__Pseudomonadota, c__Gammaproteobacteria, o__Burkholderiales, f__TRA3-20, *g__Incertae_Sedis*	3.83	0.08	Module hubs
S	ASV20684	p__Gemmatimonadota, c__Gemmatimonadia, o__Gemmatimonadales, f__Gemmatimonadaceae, *g__norank*	7.87	0.13	Module hubs
	ASV500	p__Pseudomonadota, c__Alphaproteobacteria, o__Caulobacterales, f__Caulobacteraceae, *g__Phenylobacterium*	2.85	0.00	Module hubs
	ASV15550	p__Pseudomonadota, c__Alphaproteobacteria, o__Elsterales, f__Incertae_Sedis, *g__Incertae_Sedis*	3.08	0.12	Module hubs
	ASV29887	p__Myxococcota, c__Polyangiia, o__Haliangiales, f__Haliangiaceae, *g__Incertae_Sedis*	2.85	0.00	Module hubs
	ASV32331	p__Acidobacteriota, c__Blastocatellia, o__Pyrinomonadales, f__Pyrinomonadaceae, *g__Incertae_Sedis*	1.89	0.65	Connectors

Treatment	Sequence	Taxa	Z_i	P_i	Roles
	ASV49909	p__Acidobacteriota, c__Acidobacteriae, o__Solibacterales, f__Solibacteraceae, *g__Candidatus_Solibacter*	0.66	0.63	Connectors
	ASV12951	p__Pseudomonadota, c__Gammaproteobacteria, o__Burkholderiales, f__Nitrosomonadaceae, *g__MND1*	0.06	0.66	Connectors
	ASV15188	p__Acidobacteriota, c__Blastocatellia, o__Blastocatellales, f__Blastocatellaceae, *g__Incertae_Sedis*	-1.12	0.64	Connectors
	ASV36509	p__Pseudomonadota, c__Gammaproteobacteria, o__Burkholderiales, f__SC-I-84, *g__Incertae_Sedis*	-0.53	0.80	Connectors
