# Supplementary material for: Straw-Enhanced Soil Bacterial Robustness via Resource-Driven Niche Dynamics in Tea Plantations, South Henan, China
Source: Microorganisms. 2025 Apr 6;13(4):832. doi: 10.3390/microorganisms13040832 (PMC12029857; doi:10.3390/microorganisms13040832)
Supplement: Supplementary file 1 [file microorganisms-13-00832-s001.zip › Table S7.pdf]

Table S7. The permutation test about the effects of soil physiochemical properties on the β-diversity and the keystone taxa of CK and S

Group	Soil physicochemical properties	R^2	P	P_{adj}
Bacterial β-diversity	WC	0.696	0.001	0.005
	pH	0.663	0.001	0.005
	NH_4^+-N	0.255	0.045	0.124
	AP	0.246	0.055	0.124
	SOM	0.175	0.132	0.238
	EC	0.144	0.188	0.282
	Ca	0.077	0.445	0.572
	Al	0.019	0.819	0.819
	NO_3^--N	0.017	0.815	0.819
Keystone taxa	pH	0.853	0.001	0.005
	WC	0.845	0.001	0.005
	AP	0.37	0.010	0.030
	EC	0.184	0.128	0.288
	NH_4^+-N	0.148	0.19	0.342
	SOM	0.111	0.283	0.425
	Ca	0.093	0.388	0.499
	NO_3^--N	0.011	0.882	0.932
	Al	0.006	0.932	0.932
